# Supplementary material for: Stability of Myeloid Cell Phenotype and Function Across a Broad Age Range in Humans and Cynomolgus Monkeys, and a Dominant Contribution of Humoral Factors in the Control of Bacterial Infection
Source: Biomedicines. 2025 Dec 29;14(1):71. doi: 10.3390/biomedicines14010071 (PMC12838355; doi:10.3390/biomedicines14010071)
Supplement: Supplementary file 1 [file biomedicines-14-00071-s001.zip › Supplementary Figures.pdf]

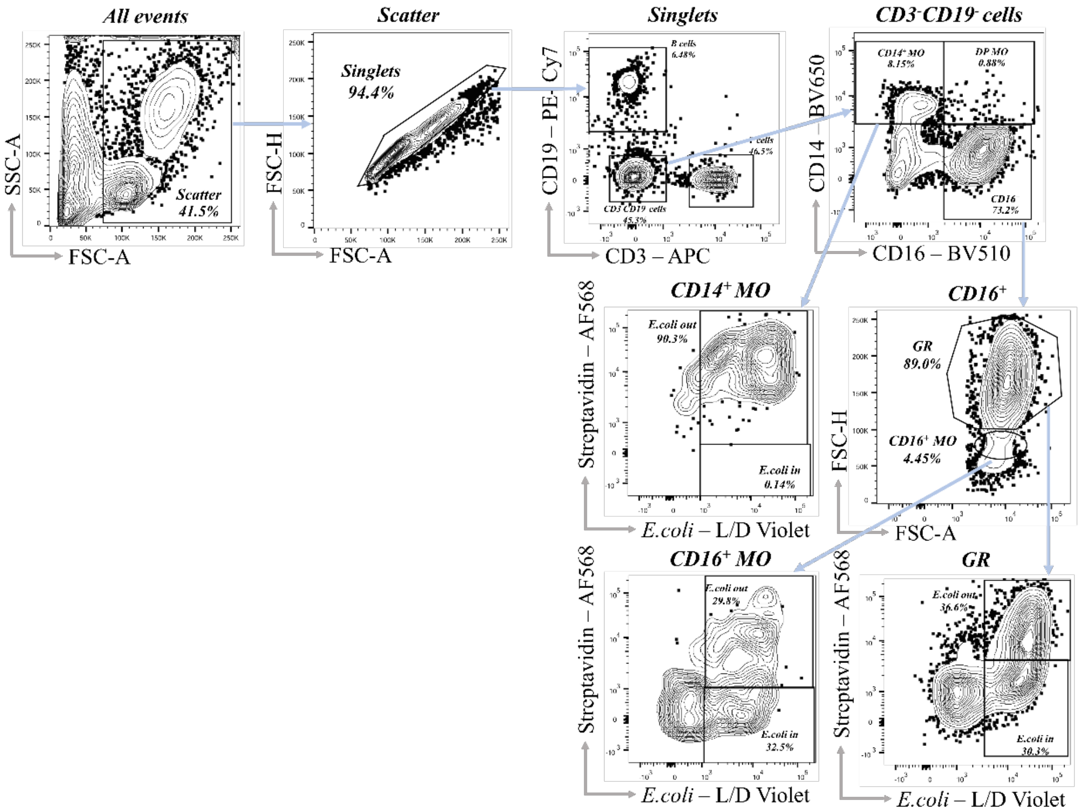

Figure S1. Gating strategy for human blood samples.

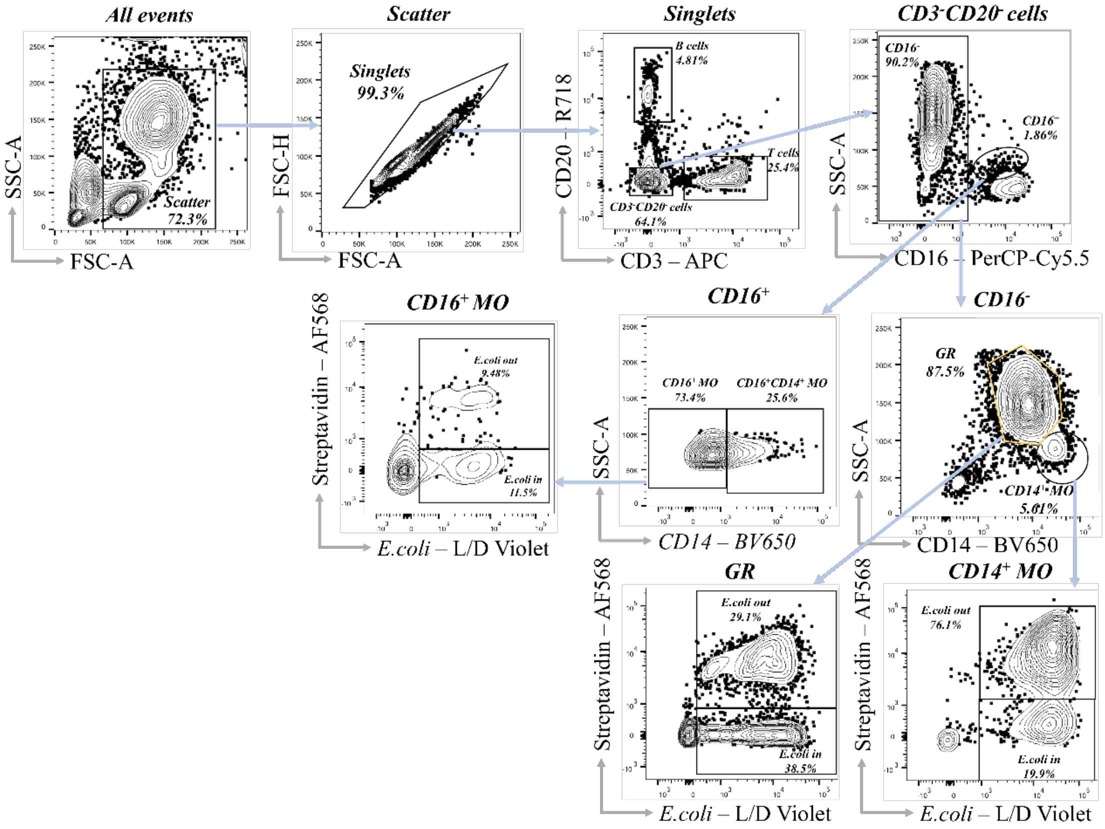

Figure S2. Gating strategy for cynomolgus monkey blood and bone marrow samples.

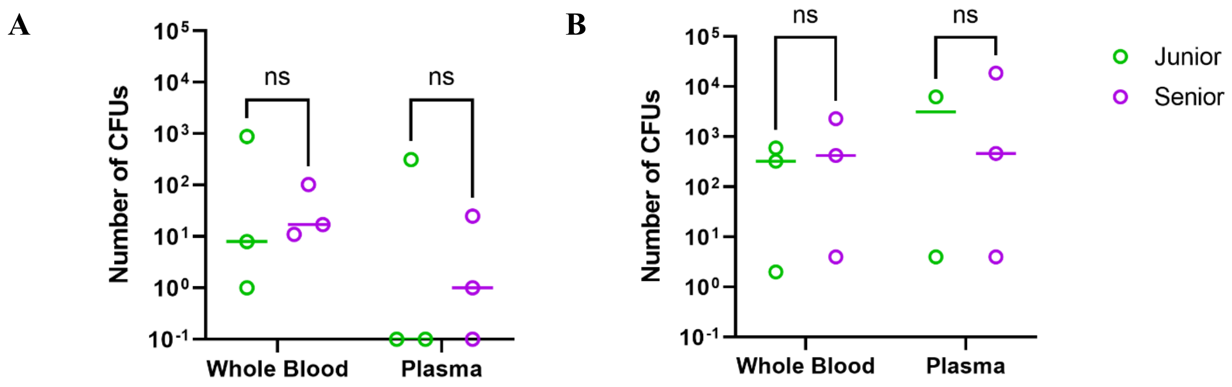

Figure S3. Comparison of antibacterial properties of whole blood and plasma between donors in two age groups (Junior and Senior). Experimental setup: A) whole blood and blood plasma were incubated for 4 hours with live bacteria without the addition of killed *E. coli*. B) samples were pre-incubated for 4 hours with fixed bacteria and then for 4 hours with live bacteria. All data are normalized to 10^8 live *E. coli* input. CFU-B counts are presented on a logarithmic scale. Statistical analysis was performed using multiple Mann-Whitney test.
