# Supplementary material for: Stability of Myeloid Cell Phenotype and Function Across a Broad Age Range in Humans and Cynomolgus Monkeys, and a Dominant Contribution of Humoral Factors in the Control of Bacterial Infection
Source: Biomedicines. 2025 Dec 29;14(1):71. doi: 10.3390/biomedicines14010071 (PMC12838355; doi:10.3390/biomedicines14010071)
Supplement: Supplementary file 1 [file biomedicines-14-00071-s001.zip › Supplementary Tables.pdf]

Table S1. Information on the age, gender and number of donors participating in the experiments.

<table>
<tr><th colspan="12">Flow cytometry</th></tr>
<tr><th colspan="4">Human blood</th><th colspan="4">M.fascicularis blood</th><th colspan="4">M.fascicularis bone marrow</th></tr>
<tr><th>Donor number</th><th>Date of sample collection</th><th>Year of birth, age</th><th>Gender</th><th>Donor number</th><th>Date of sample collection</th><th>Year of birth, age</th><th>Gender</th><th>Donor number</th><th>Date of sample collection</th><th>Year of birth, age</th><th>Gender</th></tr>
<tr><td>61</td><td>2024-02-16</td><td>1962, 61</td><td>M</td><td>35126</td><td>2024-10-08</td><td>2003, 21</td><td>M</td><td>35484</td><td>2024-11-05</td><td>2004, 20</td><td>F</td></tr>
<tr><td>62</td><td>2024-03-19</td><td>1954, 69</td><td>M</td><td>35739</td><td>2024-10-08</td><td>2004, 20</td><td>M</td><td>35800</td><td>2024-11-05</td><td>2004, 20</td><td>F</td></tr>
<tr><td>605</td><td>2024-03-19</td><td>1960, 63</td><td>M</td><td>35484</td><td>2024-10-29</td><td>2004, 20</td><td>F</td><td>35126</td><td>2024-11-14</td><td>2003, 21</td><td>M</td></tr>
<tr><td>615</td><td>2024-09-06</td><td>1954, 69</td><td>F</td><td>35763</td><td>2024-10-29</td><td>2004, 20</td><td>F</td><td>35739</td><td>2024-11-14</td><td>2004, 20</td><td>M</td></tr>
<tr><td>[616]</td><td>2024-09-06</td><td>1957, 66</td><td>F</td><td>35800</td><td>2024-10-29</td><td>2004, 20</td><td>F</td><td>278</td><td>2024-09-03</td><td>2019, 4</td><td>M</td></tr>
<tr><td>216</td><td>2024-02-16</td><td>2002, 22</td><td>F</td><td>[63 (35763)]</td><td>2024-08-21</td><td>2004, 20</td><td>F</td><td>773</td><td>2024-09-03</td><td>2019, 5</td><td>M</td></tr>
<tr><td>22</td><td>2024-03-19</td><td>1999, 25</td><td>F</td><td>[32635]</td><td>2024-08-27</td><td>1997, 27</td><td>M</td><td>47147</td><td>2024-11-05</td><td>2020, 4</td><td>F</td></tr>
<tr><td>218</td><td>2024-03-19</td><td>2000, 23</td><td>F</td><td>[35908]</td><td>2024-08-27</td><td>2004, 19</td><td>M</td><td>47167</td><td>2024-11-05</td><td>2020, 4</td><td>F</td></tr>
<tr><td>238</td><td>2024-09-06</td><td>2000, 24</td><td>F</td><td>46485</td><td>2024-10-08</td><td>2020, 4</td><td>M</td><td>47100</td><td>2024-11-14</td><td>2020, 4</td><td>M</td></tr>
<tr><td>239</td><td>2024-09-06</td><td>1999, 24</td><td>F</td><td>46580</td><td>2024-10-08</td><td>2020, 4</td><td>M</td><td>47101</td><td>2024-11-14</td><td>2020, 4</td><td>M</td></tr>
<tr><td>[302]</td><td>2024-09-06</td><td>1991, 33</td><td>M</td><td>47890</td><td>2024-10-08</td><td>2021, 3</td><td>M</td><td></td><td></td><td></td><td></td></tr>
<tr><td></td><td></td><td></td><td></td><td>45998</td><td>2024-10-29</td><td></td><td>F</td><td></td><td></td><td></td><td></td></tr>
<tr><td></td><td></td><td></td><td></td><td>46130</td><td>2024-10-29</td><td>2019, 5</td><td>F</td><td></td><td></td><td></td><td></td></tr>
<tr><td></td><td></td><td></td><td></td><td>[46005]</td><td>2024-09-03</td><td>2019, 5</td><td>M</td><td></td><td></td><td></td><td></td></tr>
<tr><td></td><td></td><td></td><td></td><td>[43866]</td><td>2024-09-03</td><td>2017, 7</td><td>F</td><td></td><td></td><td></td><td></td></tr>
<tr><td></td><td></td><td></td><td></td><td>[46247]</td><td>2024-09-03</td><td>2019, 4</td><td>M</td><td></td><td></td><td></td><td></td></tr>
<tr><td></td><td></td><td></td><td></td><td>[30 (46130)]</td><td>2024-08-21</td><td>2019, 4</td><td>F</td><td></td><td></td><td></td><td></td></tr>
<tr><th colspan="12">RESULT</th></tr>
<tr><td colspan="4">Phenotype [samples in parentheses were analyzed only for the non-phagocytotic phenotype]:
Junior 22-33 years old (n=6)</td><td colspan="4">Phenotype [samples in parentheses were analyzed only for the non-phagocytotic phenotype]:
Junior 3-7 years old (n = 9)
Senior 19-27 years old (n =8)</td><td colspan="4">Phenotype:
Junior 4 -5 years old (n = 6)
Senior 20-21 years old (n = 4)
Phagocytosis:</td></tr>
</table>

**Senior** 61-69 years old (n = 5)<br>**Phagocytosis:**<br>**Junior** 22-25 years old (n = 5)<br>**Senior** 61-69 years old (n = 4)				**Phagocytosis:**<br>**Junior** 3-5 years old (n = 5)<br>**Senior** 20-21 years old (n = 5)				**Junior** 4 -5 years old (n = 6)<br>**Senior** 20-21 years old (n = 4)			
**HMGB1, γH2AX**											
**511**	2024-04-16	1966, 57	M								
**607**	2024-04-16	1960, 64	F								
**223**	2024-04-16	1996, 27	M								
**211**	2024-04-16	2001, 22	F								
**RESULT**											
**Junior 22-27 years old** (n = 2)<br>**Senior 57-64 years old** (n = 2)											
**Analysis of serum levels of the proinflammatory factors TNF and IL-6**											
**62**	2024	1954	M								
**605**	2024	1960	M								
**606**	2024	1961	F								
**61**	2024	1962	M								
**64**	2024	1957	F								
**508**	2024	1965	F								
**63**	2024	1963	M								
**218**	2024	2000	F								
**22**	2024	1999	F								
**220**	2024	2000	M								
**24**	2024	2001	F								
**26**	2024	2000	M								
**207**	2024	2001	M								
**217**	2024	2001	M								
**RESULT**											
**Junior 23-25 years old** (n = 7)<br>**Senior 59-70 years old** (n = 7)											

**Assessment of the bactericidal activity of plasma and whole blood**											
**61**	2025-02	1962, 62	M								
**62**	2025-03-12	1954, 70	M								
**64**	2025-02	1957	F								
**242**	2025-03-12	2000, 24	M								
**207**	2025-02	2001	M								
**RESULT**											
**Junior 23-24 years old** (n = 2)<br>**Senior 62-73 years old** (n = 3)											

Table S2. Additional information on the lifestyle, addictions, BMI and some health parameters of donors participating in the experiments.

Donor number	Year of birth	Gender	Activity	Smoking	Addiction to alcohol	Waist circumference, cm	BMI	Insulin resistance	High blood pressure	High blood cholesterol	High blood glucose	Hypothyroidism	Hyperthyroidism
615	1954	F	PNA	PNA	PNA	PNA	PNA	PNA	PNA	PNA	PNA	PNA	PNA
616	1957	F	Middle	No	No	85	22	No	No	No	No	No	Yes
64	1957	F	Middle	No	No	86	24	No	No	No	No	No	No
607	1960	F	Middle	No	No	72	21	No	No	Yes	No	IDK	IDK
606	1961	F	Middle	Yes	No	107	30	No	Yes	No	No	No	No
508	1965	F	Low	No	No	95	29	IDK	Yes	No	No	IDK	IDK
62	1954	M	Middle	No	Yes	110	30	No	Yes	Yes	No	No	No
605	1960	M	Middle	No	No	85	28	No	No	Yes	No	No	No
61	1962	M	Middle	No	No	120	34	No	Yes	No	No	No	No
63	1963	M	High	Yes	Yes	80	23	No	No	IDK	No	IDK	IDK
511	1966	M	Low	Yes	No	75	28	No	No	No	No	No	No
22	1999	F	Middle	No	No	61	20	No	No	No	No	Yes	No
239	1999	F	Low	No	No	64	21	No	No	No	Yes	No	No
218	2000	F	Middle	No	No	68	21	No	No	No	No	No	No
238	2000	F	Low	No	No	65	18	No	No	No	No	No	Yes
211	2001	F	Middle	No	No	75	25	No	No	No	No	No	No

24	2001	F	Middle	No	No	74	21	No	No	Yes	No	No	No
216	2002	F	Middle	No	No	65	19	No	No	No	No	No	No
302	1991	M	Low	Yes	Yes	103	31	No	Yes	No	No	No	No
223	1996	M	Middle	No	No	IDK	25	No	No	No	No	No	No
220	2000	M	Middle	Yes	No	78	25	No	No	No	No	No	No
26	2000	M	High	No	No	62	20	No	No	No	No	No	No
242	2000	M	Middle	No	No	80	19	No	No	No	No	No	No
207	2001	M	Low	No	No	80	25	No	No	No	No	No	No
217	2001	M	Low	No	No	92	28	No	No	No	No	No	No

NR – preferred not to answer; IDK - I don't know

Table S3. A set of antibodies for staining human blood after phagocytosis.

	Antibodies for human blood staining	Working dilution
1)	CD16 – BV510 (clone 3G8, BD Horizon, BD)	1:100
2)	CD14 – BV650 (clone M5E2, BD Horizon, BD)	1:100
3)	Streptavidin – AF568 or DL550 (both Thermo Fisher)	1:200
4)	CD19 – PE-Cy7 (clone HIB19, BD Pharmingen, BD)	1:200
5)	CD3 – APC (clone SP34-2, BD Pharmingen, BD)	1:50

Table S4. A set of antibodies for staining macaque blood after phagocytosis.

	Antibodies for macaque blood staining	Working dilution
1)	CD14 – BV650 (clone M5E2, BD Horizon, BD)	1:100
2)	CD45 – BV786 (clone D058-1283, BD Horizon, BD)	1:100
3)	CD16 – PerCP-Cy5.5 (clone 3G8 , BioLegend),	1:100
4)	CD3 – APC (clone SP34-2, BD)	1:50
5)	CD20 – R718 (clone 2H7, BD Horizon, BD)	1:100
6)	Streptavidin – AF568 (Thermo Fisher)	1:200
